# Antimicrobial Resistance of *Escherichia coli* in Dairy Calves: A 15-Year Retrospective Analysis and Comparison of Treated and Untreated Animals

**DOI:** 10.3390/ani11082328

**Published:** 2021-08-06

**Authors:** Nicoletta Formenti, Chiara Martinelli, Nicoletta Vitale, Stefano Giovannini, Cristian Salogni, Matteo Tonni, Federico Scali, Laura Birbes, Mario D’Incau, Flavia Guarneri, Paolo Pasquali, Giovanni Loris Alborali

**Affiliations:** 1 Istituto Zooprofilattico Sperimentale della Lombardia e dell’Emilia Romagna “Bruno Ubertini”, Via Bianchi 7/9, 25124 Brescia, Italy; chiara.martinelli@ats-brescia.it (C.M.); nicoletta.vitale@izsto.it (N.V.); stefano.giovannini@izsler.it (S.G.); cristian.salogni@izsler.it (C.S.); matteo.tonni@izsler.it (M.T.); federico.scali@izsler.it (F.S.); laura.birbes@izsler.it (L.B.); mario.dincau@izsler.it (M.D.); flavia.guarneri@gmail.com (F.G.); giovanni.alborali@izsler.it (G.L.A.); 2 Dipartimento di Sicurezza Alimentare, Nutrizione e Sanità Pubblica Veterinaria, Istituto Superiore di Sanità, Viale Regina Elena 299, 00161 Roma, Italy; paolo.pasquali@iss.it

**Keywords:** AMR surveillance, temporal dynamics, virulence genes, ineffective treatments, 1-week-old calves, environmental contamination

## Abstract

**Simple Summary:**

In dairy production, antimicrobial resistance (AMR) is both a health and economic issue that may lead to treatment failures and the spread of multidrug-resistant pathogens. Epidemiological and farm data on AMR are instrumental for selecting the appropriate therapy. However, such data are not always available. We investigated the AMR profile of 2612 *Escherichia coli* strains isolated from cases of calf diarrhea over a 15-year period (2002–2016). Furthermore, the AMR profiles and major virulence genes of 505 *E. coli* strains isolated from 1-week- and 2-week-old calves were examined, with a comparison made between those treated with antimicrobials (n = 406) and not treated (n = 99) as well as between the two age groups to evaluate the potential effects of treatments on AMR and pathogenicity. Resistance to tetracycline was the most common, followed by resistance to sulfamethoxazole/trimethoprim and flumequine. Treated calves showed a higher rate of AMR and virulence genes. These results highlight the risk of the frequent use of antimicrobials on calf microflora in leading to potentially ineffective treatments. A higher resistance to amoxicillin/clavulanic acid, enrofloxacin, and florfenicol was found in 1-week-old calves, suggesting the environment as a possible AMR source. In conclusion, measures such as improved hygiene in the calving pen, antimicrobial stewardship, and monitoring for resistant pathogens in manure should be promoted to prevent the spread of AMR.

**Abstract:**

The health problem of antimicrobial resistance (AMR) involves several species. AMR surveillance is essential to identify its development and design control strategies; however, available data are still limited in some contexts. The AMR profiles of 2612 *E. coli* strains isolated over a period of 15 years (2002–2016) from calf enteric cases were analyzed to determine the presence of resistance and their temporal dynamics. Furthermore, the AMR profiles and the presence of the major virulence genes of 505 *E. coli* strains isolated from 1-week- and 2-week-old calves, 406 treated with antimicrobials and 99 untreated, were analyzed and compared to investigate the potential effects of treatment on AMR and strain pathogenicity. Resistance to tetracycline (90.70%) was the most common, followed by resistance to sulfamethoxazole/trimethoprim (77.70%) and flumequine (72.10%). The significantly higher percentage of AMR and virulence gene expression recorded in treated calves, combined with the statistically higher resistance to sulfamethoxazole/trimethoprim in *E. coli* with K99, corroborates the notion of resistance being induced by the frequent use of antimicrobials, leading to treatments potentially becoming ineffective. The significantly higher resistance to amoxicillin/clavulanic acid, enrofloxacin, and florfenicol in isolates from 1-week-old calves suggests the role of the environment as a source of contamination that should be investigated further.

## 1. Introduction

Antimicrobial resistance (AMR) is a global health problem that involves both humans and animal species [1,2]. This natural phenomenon occurs when a microorganism (bacterium, fungus, virus, or parasite) becomes resistant to an antimicrobial (a loss of the efficacy of treatment) [3] due to many causes, including its excessive or inappropriate use or a lack of adequate infection prevention or vaccination programs [3]. In the field of veterinary medicine, AMR may represent both a public health concern and a zoo-economic issue for food animal production systems. Indeed, the increasing resistance in foodborne zoonotic bacteria and clinical pathogens [4], together with the issue of the frequent administration of antimicrobials in dairy production [5,6,7], have led to AMR surveillance becoming a necessity. In particular, identifying the development of resistance through analyses of longitudinal AMR data is required to provide insights into temporal relationships [8], rather than limiting the analyses of prevalence to a single point in time [8]. The results from such approaches can be useful in contributing to the design of effective and efficient control strategies [9] as well as to provide veterinarians with empirical evidence and guide them in selecting drugs for therapy [10]. Many bacteria can contribute to the complexity of the problem of antimicrobial resistance, but one of the most significant is *Escherichia coli*, a ubiquitous microorganism that can behave both commensally and as a pathogen [11,12,13,14]. In dairy farms, *E. coli* is frequently responsible for neonatal colibacillosis in calves, representing a serious health and welfare problem that can result in high mortality and morbidity, thus contributing to considerable economic losses [15,16,17,18,19]. In particular, this problem could be related to the potential ineffectiveness of treatments induced by the presence of antimicrobial resistance, especially for *E. coli* expressing the K99 fimbriae and the heat-stable type Ia (STa) toxin, which is one of the major pathogens associated with this disease [20,21].

These concerns should be investigated in the field of bovine production, particularly in Italy for the long tradition and the richness of animal husbandry [22], in light of the potential negative impact that these issues may have on meat productions, similarly to what occurs for pigs [23]. Moreover, further information regarding the presence and trend of AMR in Italy needs to be gathered in light of it being one of the countries with the highest antimicrobial use in the EU [5]. Therefore, the antimicrobial resistance profiles of a total of 2612 *E. coli* strains isolated over a period of 15 years (2002–2016) from calf enteric clinical cases were analyzed to determine the presence of AMR and the potential temporal dynamics during the study years. In addition, the antimicrobial resistance profiles and the presence of the virulence genes in 505 *E. coli* strains from 1- and 2-week-old calves, either treated with antimicrobials or not treated, were analyzed and compared in order to investigate the potential effects of the treatments on both AMR and strain pathogenicity.

## 2. Materials and Methods

### 2.1. Sampling

Over a 15-year period (2002–2016), the presence of *E. coli* strains was investigated in 12,351 biological materials (carcasses, feces, fecal swabs, and intestines) from diarrheic calves aged between 1 day and 2 weeks old, as part of the routine activity of the Diagnostic Section of IZSLER in Brescia, Italy. When carcasses were available, fecal samples for diagnostic investigations were collected directly from the rectal ampulla during the necropsy following specific standard guidelines. Over the 12,351 sampling 2612 *E. coli* strains were isolated, and these strains were included in the study. The biological materials, from which *E. coli* were isolated, belonged from 607 farms located in northern Italy which had similar characteristics and a known history of the occurrence of diarrhea in calves.

From the overall 2612 isolates, 406 *E. coli* strains isolated from 406 biological samples (Table 1) of diarrheic calves, housed in 220 farms, were selected since antimicrobial treatments were known and had been performed prior to sampling and delivery to our department. Under the supervision of the veterinarians, all these calves individually received a therapeutic antimicrobial treatment with sulfonamides, fluoroquinolones, tetracycline, or oxytetracycline at the onset of diarrhea symptoms. Information about the treatments was obtained through collaboration with farm veterinarians who followed the clinical cases. For 192 out of the 406 calves, the age at sampling was known. Samples collected from such calves were grouped into two age classes: 63 from the 1-week-old calf class and 129 from the 2-week-old calf class. For the remaining 214 calves, accurate data regarding age at sampling were not available.

In addition, during 2017, a total of 99 fecal samples were collected for research purposes individually and directly from the rectal ampulla from 99 healthy calves without any clinical symptoms who had not been treated with antimicrobials. From them, a total of 99 *E. coli* strains were isolated (Table 2). These samples were collected from eight farms in which cases of calf diarrhea occurred/recurred. Sample size of treated and untreated calves was calculated by G*Power 3.1 using the formula for logistic regression (Z test family) setting an odds ratio of 2, the probability of an event under H0 as 0.1, for a two-tailed test with a significance level of 5% and a power of 80% [24].

All the samples were immediately stored at 4.0 °C until laboratory analysis.

### 2.2. Identification of E. coli

The isolation procedure was consistent during the whole study period (2002–2017). The samples were processed within 24 h after collection, cultured on both MacConkey agar plates and blood agar plates (Oxoid, Garbagnate Milanese, Milan, Italy), and incubated aerobically for 18 ± 2 h at 37 ± 2 °C. After overnight incubation, suspicious *E. coli* colonies were identified by morphology (pink on MacConkey, hemolysis on blood agar plates) and Gram staining. For each case or animal, one suspected colony with biochemical properties (lactose and indole positive; H_2_S, oxidase, and urease negative) were subcultured on brain heart infusion (BHI) agar slants (Oxoid, Garbagnate Milanese, Milan, Italy), while identities were confirmed using the API 20E biochemical method (bioMérieux, Marcy l’Etoile, France).

### 2.3. Molecular Characterization

*E. coli* samples isolated from treated and untreated calves were screened by multiplex PCR for the presence of the major virulence genes of pathogenic *E. coli*, including genes for five different adhesins (*K88*, *K99*, *F41*, *987P*, and *F18*) and four different toxins (*LT*, *STaP*, *STb*, and *Stx2e*), according to the method of Casey and Bosworth [25]. Briefly, DNA was obtained from each *E. coli* isolate (one colony) using a hot lysis procedure in which the sample was harvested by centrifugation (12,000× *g* for 5 min), washed three times in distilled water, boiled at 97.5 ± 2.5 °C for 10 min, and immediately cooled on ice for 2 min. After centrifugation, the extracted DNA was subjected to multiplex PCR to screen virulence factors (VFs) using specific primers [25]. According to [25], the PCR reaction mixtures contained 18 primers at a concentration of 0.5 µmol each, with 0.2 mmol deoxyribonucleotide triphosphate mix, 1× reaction buffer, 5 mmol MgCl_2_, and 2.5 units of Taq polymerase in a final volume of 20 µL. The amplification conditions were as follows: initial denaturation at 94 °C for 10 min, followed by 30 cycles of denaturation for 30 s at 94 °C, annealing at 55 °C for 45 s, and extension for 1.5 min at 72 °C. The extension time was increased by 3 s each cycle, and the final extension was 10 min at 72 °C. The amplification products were then separated and detected by electrophoresis [25].

### 2.4. Antimicrobial Susceptibility Testing

The susceptibility of *E. coli* strains to a panel of antimicrobials was tested using the disc diffusion method following the procedures of the Clinical and Laboratory Standards Institute [26,27]. Briefly, the isolates were inoculated in trypticase soy broth (TSB) and then plated on Mueller-Hinton agar using the following seven types of commercially available discs containing different antimicrobials: amoxicillin/clavulanic acid (AMC: 30 µg), enrofloxacin (ENR: 5 µg), florfenicol (FFC: 30 µg), flumequine (FQ: 30 µg), gentamicin (GEN: 10 µg), tetracycline (TET: 30 µg), and sulfamethoxazole/trimethoprim (SXT: 1.25/23.75 µg). The plates were read after incubation under aerobic conditions at 37 ± 2 °C for 18 ± 2 h. The isolates were classified as resistant, susceptible, or intermediate in response to the antimicrobials tested according to the zone diameter interpretative standard recommendations of CLSI [28,29,30,31,32]. Intermediate isolates were grouped with resistant isolates forming the “non-susceptible” group [27,33,34,35,36]. The complete panel of all seven antimicrobials has been used for *E. coli* collected since 2006, with some antimicrobials not available prior to this date; therefore, data gathered before 2006 are shown purely for descriptive purposes.

### 2.5. Statistical Analyses

Concerning the retrospective analyses, the prevalence of resistance was calculated for each antimicrobial and study year. Variations in the percentage of resistance between years and antimicrobials were investigated using the Kruskal–Wallis nonparametric test. The mean resistance between years was calculated using the F-test. The occurrence of resistance to at least 1 agent in 3 antimicrobial classes [37] (multidrug resistance MDR) was investigated for each isolate. To evaluate the multidrug resistance trends over the years, an analysis of variance was performed considering the incidence of multidrug resistance as the response variable and the year as the explanatory variable. Tetrachoric correlation was used to evaluate the relationship between the seven antimicrobials.

Concerning the comparison between treated and untreated calves, the prevalence of resistance was calculated for each antimicrobial, treatment group (treated and untreated calves), and age category (1-week-old and 2-week-old calves). The 214 calves for which information about their exact ages was missing were not categorized into either age group and, therefore, were not included in the statistical analysis when the explanatory variable “age” was considered. The comparison between groups was performed using the χ^2^ statistical association test. The between-group comparisons of the mean resistance percentages were carried out using t-tests, and the median percentages were compared using the Wilcoxon test. For each *E. coli* virulence gene, comparisons between groups were performed using Fisher’s exact test for the presence of low frequencies. For each isolated *E. coli* strain, the resistance developed against multiple antimicrobials was assessed by calculating the incidence of simultaneous resistance to multiple antimicrobials. The mean difference between the multidrug resistance in the treated and untreated groups was tested using the t-test, while the Wilcoxon test was used to calculate the median difference. Multivariate logistic regression models were used to investigate the factors associated with antimicrobial resistance. In particular, each antimicrobial was considered as a response variable, while treatment groups (treated and untreated), age categories (1-week and 2-week-old calves), and virulence genes (K99, F41, and StaP) were assessed as explanatory variables. The likelihood ratio test was used to assess the model’s statistical significance, while the significance of each factor was estimated using Wald’s χ^2^ test. The Hosmer–Lemeshow goodness-of-fit test was used to evaluate the model’s good fit to the data [38].

All analyses were conducted using R 3.4.0 [39]. A result was considered statistically significant when *p* < 0.05.

## 3. Results

The overall percentage of resistance to amoxicillin/clavulanic acid (AMC), enrofloxacin (ENR), florfenicol (FFC), flumequine (FQ), gentamicin (GEN), tetracycline (TET), and sulfamethoxazole/trimethoprim (SXT) of the 2612 *E. coli* strains was calculated considering data from the entire study period (2002–2016) (Table 3). A significant difference (KW 77.7, *p* < 0.0001) between these percentages was recorded (Table 3).

**Figure 1:**
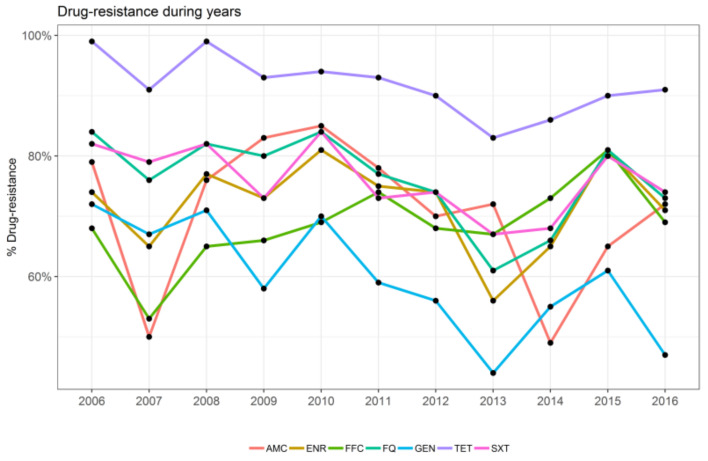
Representation of the trend of resistance for each antimicrobial over the study years as indicated: amoxicillin/clavulanic acid (AMC), enrofloxacin (ENR), florfenicol (FFC), flumequine (FQ), gentamicin (GEN), tetracycline (TET), and sulfamethoxazole/trimethoprim (SXT).

The trends of the percentage of resistance observed at the single-molecule level are reported in Figure 1. Statistically significant differences between years were recorded for FQ (F 15.43, *p* < 0.01), GEN (F 5.89, *p* < 0.05), SXT (F 13.5, *p* < 0.01), and TET (F 18.25, *p* < 0.001).

**Figure 2:**
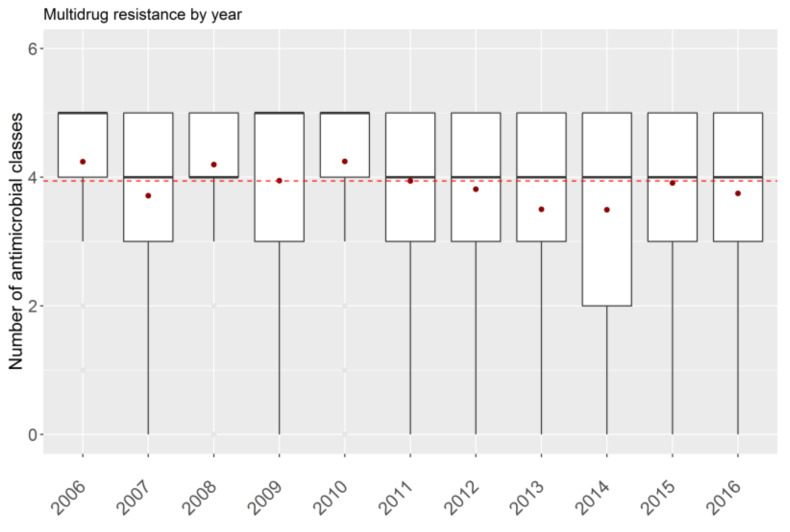
Trend of multidrug resistance during the study period. Red dashed line: the mean value of antimicrobial classes calculated for the whole period; red dots: mean value calculated for each year.

Concerning multidrug resistance, the recorded mean resistance was 4.33 (SD = 0.75) for antimicrobial classes, while the median value was 5. A significant difference in multidrug resistance was observed during the study years (test F 12.8, *p* < 0.001) (Figure 2).

**Figure 3:**
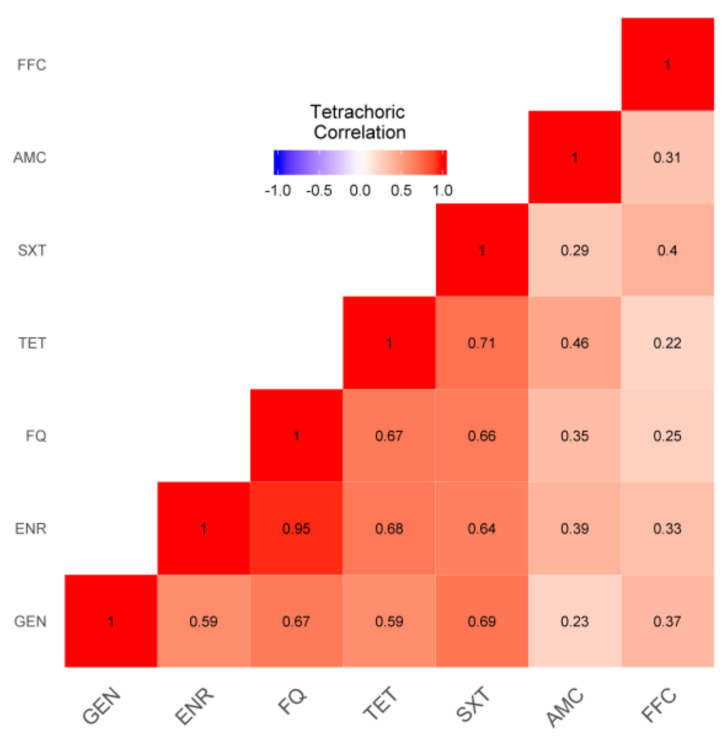
Heat map of the associations between antimicrobials. Red indicates positive associations, with the intensity of red shading corresponding to the strength of the association. Amoxicillin/clavulanic acid (AMC), enrofloxacin (ENR), florfenicol (FFC), flumequine (FQ), gentamicin (GEN), tetracycline (TET), and sulfamethoxazole/trimethoprim (SXT).

Regarding the associations between resistances to antimicrobials, positive correlations were recorded between FQ and ENR (0.95), SXT and TET (0.72), SXT and GEN (0.69), TET and ENR (0.68), GEN and TET (0.67), FQ and TET (0.67), and GEN and FQ (0.67) (Figure 3). No negative patterns were observed (Figure 3).

The percentage of resistance to each antimicrobial was calculated for *E. coli* isolated from both untreated and treated calves (Table 4). The percentage of resistance was significantly higher in the treated group than in the untreated group for all antimicrobials except TE (Table 4).

The prevalence of virulence genes recorded in *E. coli* isolated from both treated and untreated calves is reported in Table 5. The presence of *K99*, *F41*, and *StaP* was significantly higher in *E. coli* isolated from treated calves than in isolates from untreated calves (*p* < 0.05, Table 5). *Stx2e* was more prevalent in *E. coli* isolated from untreated than in treated calves, but the relationship was not significant (*p* = 0.054).

**Figure 4:**
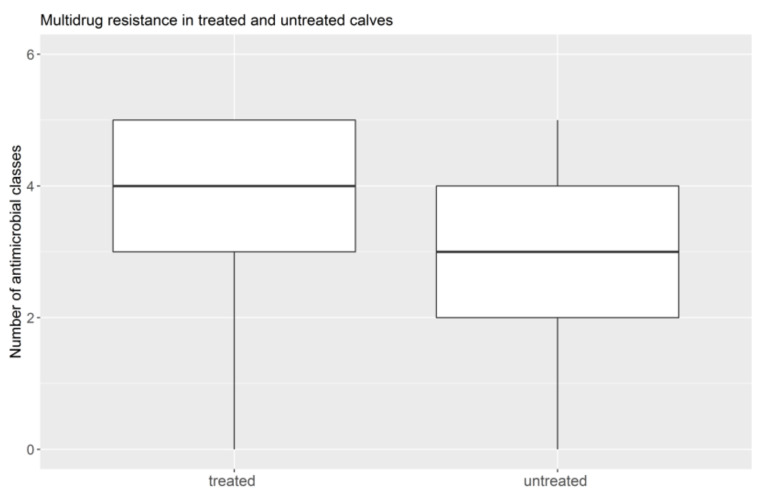
Distribution of multidrug resistance among the two treatment groups of *E. coli*, isolated from either treated or untreated calves.

Six strains were resistant to all seven antimicrobials, and they were all isolated from treated calves. In 30 *E. coli* strains (18 isolated from treated calves and 12 from untreated calves), no resistance to any antimicrobial was recorded. Concerning MDR, the isolates from untreated calves showed a lower incidence, with a median value of 3 antimicrobials and a mean of 2.7 (Figure 4). The median and mean value of multidrug resistance of *E. coli* from treated calves was 4 antimicrobials in both cases (Figure 4). The differences were significant for both the means of the multidrug resistance (t 9.5, *p* < 0.0001) and for the median values (W 66.05, *p* < 0.0001).

Concerning logistic regression for AMC, ENR, and FFC, a significant difference between the resistance of *E. coli* isolated from 1- versus 2-week-old calves emerged (Table 6). A higher probability of recording resistance to these antimicrobials was observed in 1- than in 2-week-old calves. For FQ, GEN, SXT, and TET, a higher probability of recording resistance to these antimicrobials was observed in the treated than in the untreated calves (Table 6). A significant association between SXT and K99 was recorded (Table 6). In particular, the probability of recording resistance to for SXT antimicrobial in *E. coli* harboring *K99* was two times higher (OR 2.01) than in *E. coli* without *K99*.

## 4. Discussion

This study highlights the occurrence of antimicrobial resistance in *E. coli* isolated from diarrheic calves over a period of 15 years (2002–2016). Resistance to TET, SXT, and FQ occurred most frequently. Calves treated with antimicrobials showed a significantly higher percentage of antimicrobial resistance and a significantly higher expression of virulence genes (*K99*, *F41*, and *StaP*) than untreated calves. Moreover, the detection of significantly higher resistance to AMC, ENR, and FFC in *E. coli* isolated from 1-week-old than in strains from 2-week-old calves suggests the role of the environment as a contamination source.

Resistance to all the considered antimicrobials was recorded, although the highest percentage was registered for TET (90.4%), SXT (77.70%), FQ (72.10%), and GEN (59.20%). These results are consistent with those of previous surveys and are likely due to their extensive therapeutic use in cattle [40,41,42]. Moreover, in light of the high prevalence of tetracycline resistance, Authors [43,44] have suggested the significant role of lactating cattle as reservoirs of tetracycline-Gram-negative enteric bacteria [45]. As previously reported, the mean number of drugs for which multidrug resistance was recorded was four, with a median value of five [8,44,46]. Analysis of the correlation between antimicrobial resistance in *E. coli* isolates showed that the highest tetrachoric correlation was between FQ and ENR (0.95). In other words, the presence of resistance to FQ, resistance to ENR was also expected. Both these antimicrobials belong to the class of fluoroquinolones, which have excellent activity against *E. coli*. This result leads to the hypothesis that their frequent use may favor co-selection [47], which may also be ascribed to their similar chemical structures [48]. In addition, the high tetrachoric correlation that emerged between resistance to SXT and TET (0.72) could be due to their common use in calf treatments [5,49].

Concerning both the results of the χ^2^ association and those of logistic regression, the percentage of resistance was significantly higher in *E. coli* isolated from treated calves than in the untreated group for all antimicrobials. This evidence supports the notion that the use of antimicrobials has resulted in selection pressure on the calves’ gastrointestinal bacteria and favored the presence of resistant strains [45,50,51]. In addition, significantly higher multidrug resistance values were recorded in *E. coli* samples taken from treated calves, compared to untreated calves, with a median value of four antimicrobials. Nevertheless, multidrug resistance was recorded even in strains from the untreated group, although this was lower in terms of both the percentage of affected calves and the number of drugs for which resistance was demonstrated, suggesting that resistance was established regardless of use. This result can be attributed to the transmission of resistance bacteria through contaminated environments and the sharing of spaces with older animals that have undergone antimicrobial treatments [45]. The fact that virulence genes (*K99*, *F41*, and *StaP*) were significantly more prevalent in isolates from treated than in untreated calves leads to the hypothesis that antimicrobial therapeutic treatment plays a role in the selection of pathogenic strains.Otherwise, the alternative hypothesis that the presence of more pathogenic *E. coli* requires more therapeutic antimicrobial treatments, and therefore virulence genes were significantly more prevalent in isolates from treated calves, cannot be ruled out. In any case, the higher probability of recording resistance to SXT in *E. coli* expressing K99 supports previous hypotheses and all these results lead to questions about the future effectiveness of treatments for such pathogenic *E. coli*. Despite the methodological bias of comparing 406 strains isolated from treated calves over a period of 15 years, and 99 *E. coli* detected from untreated calves during only one sampling year, including the negative sampling allowed the use of these valuable data, which emerged over 15 years from treated calves whose all the clinical information and antimicrobial treatments were available and highlighted the effects of treatments on the presence of AMR, which was the aim of this study. Moreover, the considerable numerical difference between the sample size of treated and untreated calves was due to the difficulties in planning and conducting the sampling of healthy, untreated calves (n = 99), especially concerning the selection of farms from which the sampling was allowed. However, although there were methodological limitations, our results were consistent with those of previous studies on the role of the over-use of antimicrobials in favoring the selection pressure for resistant bacteria, supporting the drawn conclusions.

The significantly higher probability of *E. coli* isolated from 1-week-old calves being resistant to AMC, ENR, and FFC could be related to the known decline in susceptibility to resistance with the increase in age [45,52,53]. Moreover, as the introduction of resistant bacteria into the enteric microbiota depends on their ability to compete with indigenous microbiota effectively, younger calves lack a developed and diverse intestinal microflora, which could reduce their degree of protection against bacterial colonization [54]. In addition, this result suggests that 1-week-old calves could have acquired this resistance directly from dams [53] or through the farm environment [6]. Indeed, the role of environmental contamination, mainly from the calving pen, as a vehicle for AMR bacteria transfer or an antimicrobial contamination source [55,56], should be further investigated. Furthermore, the fecal shedding of these strains and their long-term survival in manure or in the environment [30,57] should be taken into account.

## 5. Conclusions

This study aimed to obtain long-term data on AMR in *E. coli* from calves over 15 years of study and to evaluate the potential effects of antimicrobial treatments and the differences in the resistance and pathogenicity of strains in subjects of different age classes. The *E. coli* isolated from calves treated with antimicrobials showed significantly higher levels of antimicrobial resistance and a two-times higher probability of recording resistance to SXT in *E. coli* with *K99* than in strains without *K99*. The detection of significantly higher resistance to AMC, ENR, and FFC in *E. coli* isolated from 1-week-old calves than in strains isolated from 2-week-old calves suggests an in-depth analyses of the role of the farm environment as a source of AMR bacteria contamination. Improved hygiene in the calving pen could considerably reduce the risk of bacterial contamination. Further investigations regarding resistant pathogens in manure are needed to elucidate the potential health risks involved.

## Figures and Tables

**Table 1:** Description of the 406 biological materials from which the *E. coli* strains were isolated.

Age Group	N° Feces/Fecal Samples from Rectal Ampulla	N° Fecal Swabs	N° Intestines	Total
1 week old	60	0	3	63
2 weeks old	115	6	8	129
Not available	205	4	5	214
Total	380	10	16	406

**Table 2:** Overview of the sampling of untreated calves sorted by farm and age class.

Farms	1 Week Old	2 Weeks Old	Total
1	3	0	3
2	5	11	16
3	0	2	2
4	6	7	13
5	2	13	15
6	4	20	24
7	10	0	10
8	10	6	16
Total	40	59	99

**Table 3:** Total number of resistant isolates and percentage of resistance over the entire study period for each antimicrobial. Amoxicillin/clavulanic acid (AMC), enrofloxacin (ENR), florfenicol (FFC), flumequine (FQ), gentamicin (GEN), tetracycline (TET), sulfamethoxazole/trimethoprim (SXT).

Antimicrobials	N° of Resistant Isolates	Total	Percentage of Resistance	LCI95%	UCI95%
AMC	879	2126	41.30%	40.30%	42.40%
FFC	968	1968	49.20%	48.10%	50.30%
GEN	1534	2591	59.20%	58.30%	60.10%
ENR	1550	2438	63.60%	62.70%	64.50%
FQ	1877	2603	72.10%	71.30%	72.90%
SXT	2024	2605	77.70%	77.00%	78.40%
TET	2354	2595	90.70%	90.40%	91.00%

**Table 4:** Percentages of resistance to each antimicrobial in *E. coli* according to isolation from treated or untreated calves. Amoxicillin/clavulanic acid (AMC), enrofloxacin (ENR), florfenicol (FFC), flumequine (FQ), gentamicin (GEN), tetracycline (TET), sulfamethoxazole/trimethoprim (SXT).

Antimicrobials	Percentage of Resistance of *E. coli* from Untreated Calves	Percentage of Resistance *E. coli* from Treated Calves	χ^2^	*p*
AMC	52.53% (52/99)	82.18% (332/404)	37.087	0.0000
ENR	39.39% (39/99)	75.19% (303/403)	45.255	0.0000
FFC	44.44% (44/99)	70.28% (253/360)	21.573	0.0000
FQ	39.39% (39/99)	80.30% (326/406)	64.427	0.0000
GEN	24.24% (24/99)	61.39% (248/404)	42.692	0.0000
SXT	44.44% (44/99)	75.80% (307/405)	35.533	0.0000
TET	88.89% (88/99)	94.80% (383/404)	3.7272	0.0535

**Table 5:** Prevalence of virulence genes, adhesins K99, F41, F18 and toxins LT (heat-labile), STaP, STb (heat-stable) and Stx2e (Shiga toxin), recorded in *E. coli* isolated from treated and untreated calves.

*E. coli* Virulence Genes	Prevalence in *E. coli* from Untreated Calves	Prevalence in *E. coli* from Treated Calves	*p*
K99	3.03% (3/99)	21.67% (88/406)	0.001
F41	3.03% (3/99)	11.33% (46/406)	0.012
F18	0.00% (0/99)	0.49% (2/406)	0.999
LT	0.00% (0/99)	0.49% (2/406)	0.999
StaP	3.03% (3/99)	22.91% (93/406)	0.001
STb	0.00% (0/99)	0.74% (3/406)	0.999
Stx2e	4.04% (4/99)	1.01% (4/397)	0.054

**Table 6:** List of factors found by multivariate logistic regression models that significantly influence the resistance of each antimicrobial. Amoxicillin/clavulanic acid (AMC), enrofloxacin (ENR), florfenicol (FFC), flumequine (FQ), gentamicin (GEN), tetracycline (TET), sulfamethoxazole/trimethoprim (SXT).

Antimicrobials	Factors	Baseline	OR	95% CI	LRχ2	Pr (>χ^2^)
AMC	Age category *	2 week-old	3.04	1.68–5.75	14.29	0.0001
ENR	Age category *	2 week-old	1.77	1.06–3.09	4.7	0.03
FFC	Age category *	2 week-old	2.3	1.33–4.05	9.12	0.003
FQ	Treatment group **	untreated	6.27	3.93–10.11	60.49	0.0001
GEN	Treatment group **	untreated	4.97	3.05–8.35	45.37	0.0001
TET	Treatment group **	untreated	2.28	1.03–4.81	4.08	0.043
STX	K99 ***	absence	2.01	1.21–3.32	7.16	0.007
	Treatment group **	untreated	4.56	2.84–7.38	39.91	0.0001

* age group: 1-week-old calves versus 2-week-old calves; ** treatment group: antimicrobial treated animals versus non-treated animals; *** virulence factors: presence of K99 gene in *E. coli* versus absence of K99 gene.

## Data Availability

The raw data supporting the conclusions of this article will be made available by the authors, without undue reservation.
